# Retinal vessel shift and its association with axial length elongation in a prospective observation in Japanese junior high school students

**DOI:** 10.1371/journal.pone.0250233

**Published:** 2021-04-22

**Authors:** Shotaro Asano, Takehiro Yamashita, Ryo Asaoka, Yuri Fujino, Hiroshi Murata, Hiroto Terasaki, Naoya Yoshihara, Naoko Kakiuchi, Taiji Sakamoto

**Affiliations:** 1 Department of Ophthalmology, The University of Tokyo, Tokyo, Japan; 2 Department of Ophthalmology, Kagoshima University Graduate School of Medical and Dental Sciences, Kagoshima, Japan; 3 Department of Ophthalmology, Seirei Hamamatsu General Hospital, Shizuoka, Japan; 4 Seirei Christopher University, Shizuoka, Japan; 5 Nanovision Research Division, Research Institute of Electronics, Shizuoka University, Shizuoka, Japan; 6 The Graduate School for the Creation of New Photonics Industries, Shizuoka, Japan; Faculty of Medicine, Cairo University, EGYPT

## Abstract

**Purpose:**

To investigate retinal vessel shift (RVS) and its association with axial length (AL) elongation in junior high school students.

**Methods:**

Total 161 eyes of 161 healthy junior high school students were prospectively studied. Optical AL and anterior chamber depth (ACD) measurements, and fundus photography were performed in the first and third grades. Eyes of subjects in the first and third grade that had perfect matching among all the retinal vessels were allocated to the RVS(−) group, otherwise allocated to the RVS(+) group. In the RVS(+) group, the peripapillary retinal arteries angle (PRAA) was measured for quantitative analysis of RVS; the angle between the major retinal arteries. The variables related to PRAA were identified using model selection with the corrected Akaike information criterion.

**Results:**

Forty-two eyes (26.1%) were allocated to the RVS(+) group. There were seven patterns in the RVS of those in the RVS(+) group, including clockwise shift in the supra temporal area (5 eyes), infra temporal area (7 eyes), and nasal area (9 eyes); anticlockwise shift in the supra temporal area (7 eyes), infra temporal area (5 eyes), and nasal area (2 eyes); and distal shift in the temporal area (7 eyes). The optimal model for the PRAA narrowing included larger AL and body weight in the first grade, and greater AL elongation.

**Conclusion:**

Various (seven) RVS patterns were observed in about 25% of the junior high school students within two years. RVS was associated with AL elongation, and useful to reveal the mechanism of myopic retinal stretch.

## Introduction

Myopia is one of the most prevalent ocular disorders, and high myopia is a common cause of blindness [1]. The global prevalence of myopia has increased rapidly during the previous 50 years and poses a considerable socio-economic burden on individuals and the society [2,3]. It is becoming a more serious problem in Asian countries [4–6] because the incidence is increasing and the age of the onset is lowering [2,3]. In subjects with myopia, the eyes are elongated, and consequently, in particular, the retina is stretched around the papillo-macular bundle [7]. Axial length (AL) is frequently used to estimate the elongation of an eye [8]; however, we have recently reported that the magnitude of retinal stretch cannot be fully explained with AL alone, probably due to a large variation of AL at birth across individuals [9]. One of the characteristic findings in such eyes is that the supra and infra thick retinal nerve fiber (RNF) bundles shift toward the fovea as axial elongation [7,10,11]. Our earlier study showed that retinal vessel trajectory was significantly correlated with the RNF bundle trajectory (R = 0.92) [7]. Thus, it is useful to analyze the retinal vessel trajectory to understand the retinal structural change associated with the development of myopia.

In contrast, myopic subjects have a significantly higher incidence of glaucomatous optic nerve damage [12–15], and high myopic subjects have a higher risk of various ocular diseases, such as choroidal neovascularization, retinal detachment, macular hole, and glaucoma [12,16]. Thus, investigating the detailed mechanisms of myopic change is not only important to understand the myopic retinal changes, but also to understand various ocular pathologies. For example, in the early stage of glaucoma in myopic eyes, the nerve fiber layer defect (NFLD) and corresponding visual field defects are more commonly detected in the paracentral area [17]. These defects can lead to impairments of central vision and can result in reduced visual acuity. The shift in the myopic RNF bundle and retinal vessel [retinal vessel shift (RVS)] can precede paracentral NFLDs in glaucomatous myopic eyes [18].

We hypothesize that RVS may occur in the growth phase when the eyeball is elongated. However, to our knowledge, no prospective, longitudinal study has studied the changes in RVS and AL elongation. Therefore, we aimed to prospectively observe and evaluate the RVS in junior high school students for a period of two years. Furthermore, the association between RVS and AL elongation was investigated.

## Materials and methods

### Study population

All the procedures used in this study conformed to the tenets of the Declaration of Helsinki, and they were approved by the Ethics Committee of the Kagoshima University Hospital. Written informed consent was obtained from all the subjects and their parents. This study was registered with the University Hospital Medical Network-clinical trials registry (No. UMIN000015239). This was part of a longitudinal, prospective, observational study performed on 12- or 13-year-old first-grade students of a junior high school at the Faculty of Education of Kagoshima University. There were 200 students in the third grade of elementary school, and informed consent was obtained from 178 (89.0%) students and their parents. Students were examined from January 13 to February 13, 2015 when they were in their first grade, and the same subjects were examined 2 years thereafter when they were in the third grade (age 14 or 15 years). Eight students were excluded due to incomplete or unreliable ocular examination. Four students were excluded due to truant or transfer. Using the questionnnaire sheet, fundus photography, and optical coherence tomography (OCT), subjects with previous or present eye disease or systemic disease that complicated eye disease were screened. Five eyes were excluded due to optic disc abnormality (one optic nerve atrophy, three superior segmental optic nerve hypoplasia, and one optic disc anomaly). Thus, the right eyes of 161 (80.5%) individuals were used for the analyses. Color fundus photographs were taken with the 3D OCT-1 Maestro (Topcon, Tokyo, Japan) and AL was measured with the OA-2000 Optical Biometer (Tomey, Nagoya, Japan). Only the data of the right eyes were statistically analyzed to avoid false precise confidence intervals and low *P* values.

### Classification of the RVS

**Fig 1:**
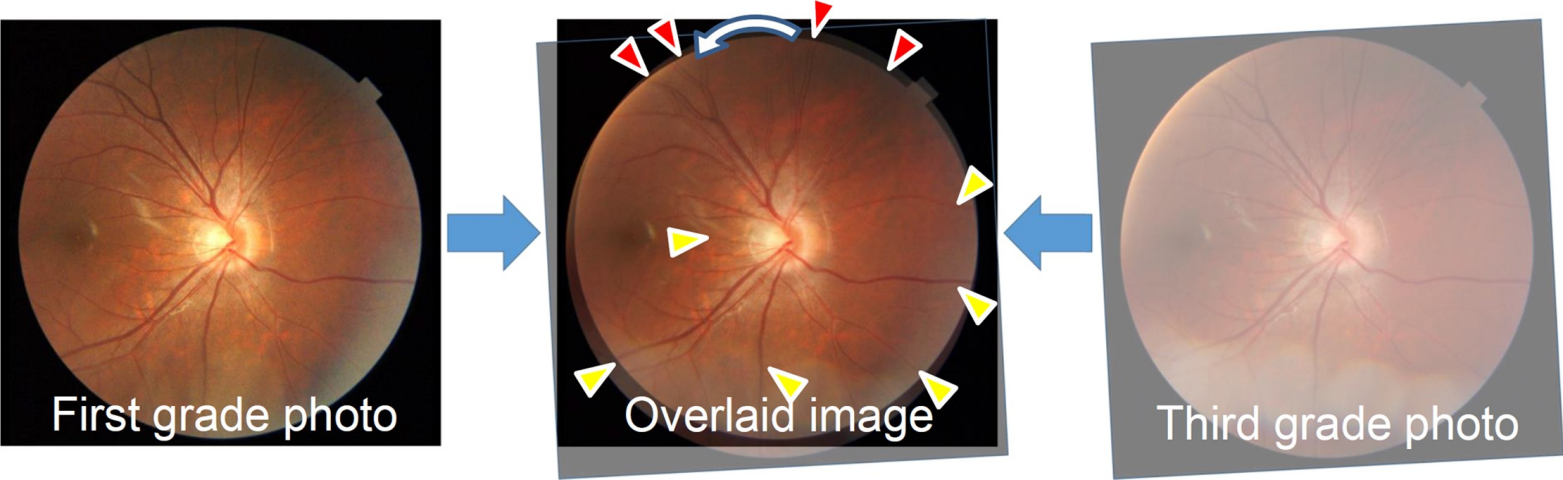
Assessment of the retinal vessel shift using fundus photographs of the subjects when in the first and third grades. Perfect matching in the temporal, inferior, and nasal vessels (yellow triangles) and mismatching (anticlockwise shift) in the superior vessels (red triangles).

**Fig 2:**
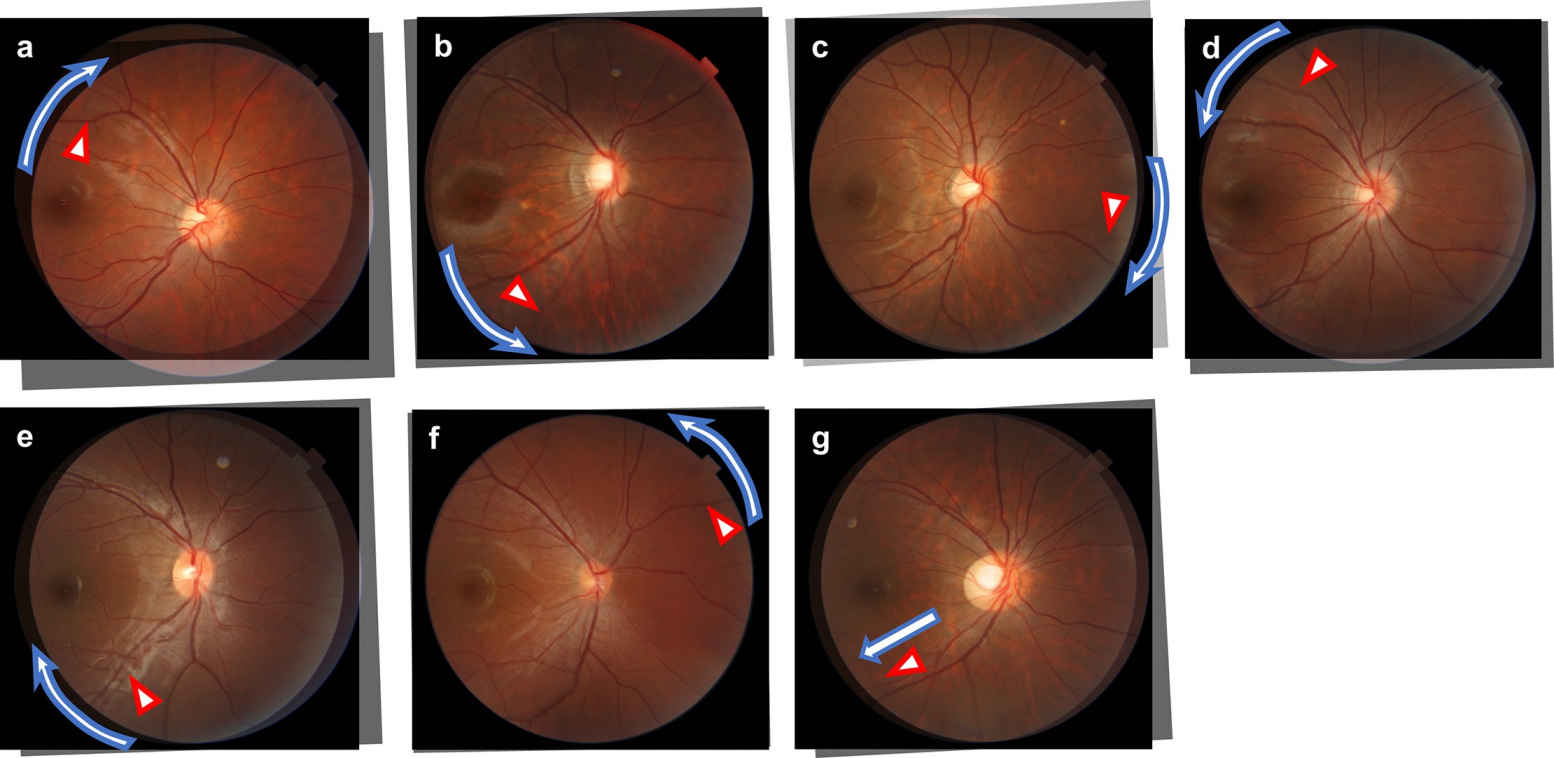
Overlaid images of several types of the retinal vessel shift. Red triangles indicate local mismatching vessels, and white arrows indicate local vessels shift. There were seven types of retinal vessel shifts: (a) supra temporal clockwise, (b) infra temporal anticlockwise, (c) nasal clockwise, (d) supra temporal anticlockwise, (e) infra temporal clockwise, (f) nasal anticlockwise, and (g) temporal distal.

RVS was assessed based on the color fundus photographs. The original fundus image that was taken at the third grade was made transparent using Photoshop software after adjusting the magnification effect with axial length [19,20]. Then, it was overlaid on the fundus image taken from the same eye when the students were in the first grade. Superimposing was done by adjusting the fine vessels on the optic disc and vessels in the temporal side of the optic disc (**Fig 1**). The fundus photographs with eyes that had perfect matching between all the retinal vessels when the subjects were in the first and third grade were categorized into the RVS(−) group, and the eyes with mismatching in some vessels were categorized into the RVS(+) group. The RVS(+) eyes were categorized into seven subgroups, supra temporal clockwise (around the optic disc), infra temporal clockwise (around the optic disc), nasal clockwise (around the optic disc), supra temporal anticlockwise (around the optic disc), infra temporal anticlockwise (around the optic disc), nasal anticlockwise (around the optic disc), and temporal distal (**Fig 2**). This classification was made using a vessel which had a most evident shift (either of artery and vein). The judgment was made by two independent examiners (TY, NK). If no consensus could be reached about the diagnosis, it was determined by the third examiner (HT).

### Peripapillary Retinal Arteries Angle (PRAA)

**Fig 3:**
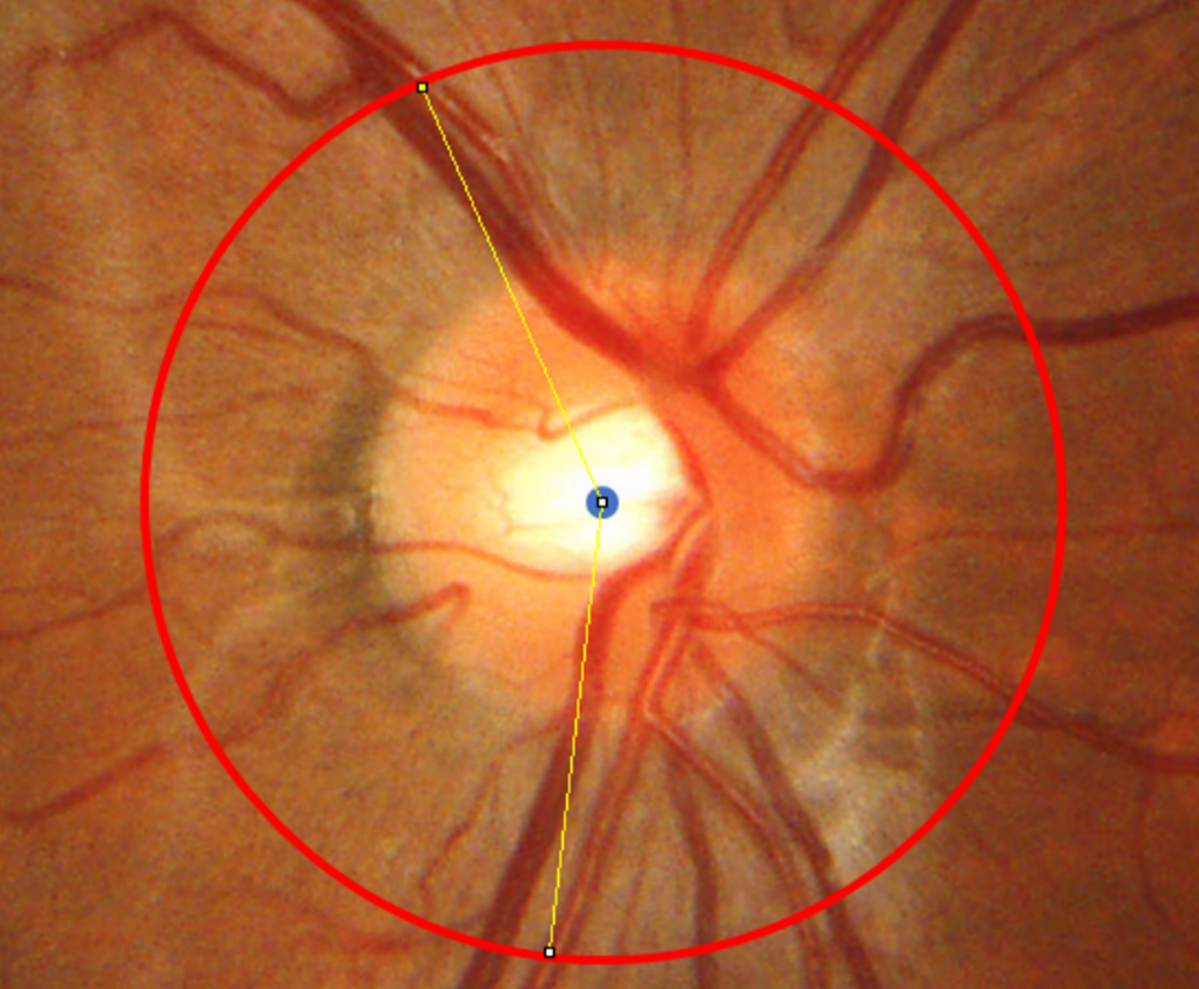
An example of the peripapillary retinal arteries angle measurement (right eye). The peripapillary retinal arteries angle (PRAA) was calculated by identifying the angle between the intersecting positions (white dots) of a 3.4-mm-diameter peripapillary scan circle (yellow) and the supratemporal/infratemporal major retinal artery.

For quantitative analyses of RVS, the peripapillary retinal arteries angle (PRAA) [7,16] was measured in the RVS(+) group. Based on the previous studies [21,22], a 3.4 mm-diameter peripapillary scan circle obtained from the OCT image was allocated on the superimposed disc color fundus photographs based on the shape of the optic disc using ImageJ software (https://imagej.nih.gov/ij/). Using the scan circle and points where the circle and the center of the superotemporal/infratemporal major retinal artery intersected, the PRAAs at first and third grade were measured respectively (**Fig 3** shows an example of a PRAA measurement).

### Statistical analyses

The Mann-Whitney U test was used to detect the statistically significance difference of body height, body weight, ACD, AL, and the difference in the body height, body weight, AL, and ACD of the subjects in the RVS(+) and RVS(−) groups at each visit when they were in the third and first grade (Δbody height, Δbody weight, ΔAL, and ΔACD, respectively). Wilcoxon signed rank tests were carried out to compare these values between the first and third grade in each group. The relationship of ΔAL with i) sex, ii) body height (first grade), iii) body weight (first grade), iv) ACD (first grade), v) AL (first grade), vi) Δbody height, and vii) Δbody weight was investigated using univariate analyses. This analysis was iterated using multivariate linear regression, followed by the model selection with round-robin method to identify the optimal model using second-order bias-corrected Akaike information criterion (AICc) index. Namely, the optimal model was selected from 2^7^ models when there were 7 explanatory variables. The AICc is a corrected version of the Akaike information criterion [23] that provides an accurate estimation even when the sample size is small [24]. The decrease in AICc indicates improvement of the model [25]. The selected variables through the model selection were regarded as significant because they provide us objective measures for selecting from among different models fitted to the data, considering the contributions and interactions among the parameters [26].

Subsequently, the following sub-analysis was conducted in the RVS(+) group. First, the PRAA values of subjects when in the first grade (age 12–13 years) and third grade (age 14–15 years) of junior high school were measured and compared using the Wilcoxon signed rank test. Then, ΔAL of positive and negative ΔPRAA eyes were compared. Moreover, the relationships between the difference in the PRAA value between the third and first grade (ΔPRAA) and the following 10 variables were investigated: i) sex, ii) body height (first grade), iii) body weight (first grade), iv) ACD (first grade), v) AL (first grade), vi) PRAA (first grade), vii) Δbody height, viii) Δbody weight, ix) ΔACD, and x) ΔAL. This analysis was also iterated using multivariate linear regression.

All the statistical analyses were performed using R (version 3.4.3, http://www.R-project.org/). P value less than 0.05 was considered to be statistically significant.

## Results

### AL and its elongation in the RVS(+) and RVS(−) groups

The background characteristics of the participants are shown in **Table 1**. Forty-two eyes (26.1%, 19 boys and 23 girls) were categorized into the RVS(+) group, and the remaining 119 eyes (60 boys and 59 girls) were allocated to the RVS(−) group. The body weight of RVS(+) group subjects in the first grade was significantly higher than that of the RVS(−) group subjects (p = 0.033, Mann-Whitney U test).

**Table 1:** Background characteristics of the participants.

	RVS(+)			RVS(−)			P Value (RVS(+) vs. RVS(−))
	Mean ± SD	Range	P Value (First vs. third grade)	Mean ± SD	Range	P Value (First vs. third grade)	
Subjects, n	42			119			
Sex (male/female)	19/23			60/59			0.69
Body height, cm							
First grade	154.42 ± 6.25	139.3–167.0	< 0.0001	153.71 ± 7.42	137.1–183.6	< 0.0001	0.25
Third grade	161.00 ± 7.28	144.8–180.9	-	160.82 ± 8.69	143.0–188.1	-	0.73
Δbody height	6.58 ± 5.29	0.5–19.6	-	7.11 ± 4.72	0.2–18.7	-	0.30
Body weight, kg							
First grade	44.68 ± 5.28	33.3–56.4	< 0.0001	42.84 ± 6.83	29.7–66.1	< 0.0001	0.033
Third grade	51.85 ± 5.76	41.3–64.5	-	51.37 ± 7.69	38.0–79.0	-	0.34
Δbody weight	7.17 ± 3.90	-1.5–16.7	-	8.53 ± 3.97	0.8–17.9	-	0.059
ACD, mm							
First grade	3.71 ± 0.24	2.90–4.37	0.0020	3.74 ± 0.24	3.07–4.38	< 0.0001	0.45
Third grade	3.73 ± 0.25	2.94–4.42	-	3.79 ± 0.24	3.29–4.43	-	0.41
ΔACD	0.02 ± 0.12	-0.62–0.16	-	0.04 ± 0.08	-0.17–0.36	-	0.52
AL, mm							
First grade	24.51 ± 1.21	21.95–26.91	< 0.0001	24.57 ± 1.24	21.91–28.94	< 0.0001	0.87
Third grade	24.81 ± 1.28	22.06–27.43	-	24.86 ± 1.30	22.05–29.20	-	0.94
ΔAL	0.30 ± 0.18	0–0.82	-	0.29 ± 0.18	-0.01–0.92	-	0.48

RVS, retinal vessel shift; SD; standard deviation, Δbody height, body height change; Δbody weight, body weight change; ACD, anterior chamber depth; ΔACD, ACD change; AL, axial length; ΔAL, AL change.

**Table 2** shows the relationships of ΔAL with body height (first grade), body weight (first grade), ACD (first grade), AL (first grade), Δbody height, and Δbody weight. The optimal linear model for ΔAL was as follows; ΔAL = -0.53 + 0.043 (SE = 0.011, p < 0.0001) × AL (first grade)– 0.0057 (SE = 0.0020, p = 0.0052) × body weight (first grade) (AICc = -117.9).

**Table 2:** The relationships of change in the AL with sex, body height (first grade), body weight (first grade), ACD (first grade), AL (first grade), Δbody height, and Δbody weight.

	Univariate analysis			Optimal model		
Variables	Coefficient	SE	*P* Value	Coefficient	SE	*P* Value
Sex	−0.0031	0.028	0.91	N.S.		
Body height (first grade)	−0.0039	0.0019	0.048	N.S.		
Body weight (first grade)	−0.0053	0.0021	0.014	−0.0057	0.0020	0.0052
AL (first grade)	0.042	0.011	< 0.001	0.043	0.011	< 0.0001
ACD (first grade)	0.096	0.057	0.094	N.S.		
ΔBody height	0.0049	0.0028	0.089	N.S.		
ΔBody weight	0.006	0.0035	0.084	N.S.		

AL, axial length; ACD, anterior chamber depth;Δbody height, body height change; Δbody weight, body weight change; SE, Standard Error; N.S., Not Selected.

### Classification of RVS

In the RVS(+) group, the pattern in 5 eyes was categorized as supra temporal clockwise (**Fig 2A**), in 5 eyes as infra temporal anticlockwise (**Fig 2B**), in 9 eyes as nasal clockwise (**Fig 2C**), in 7 eyes as supra temporal anticlockwise (**Fig 2D**), in 7 eyes as infra temporal clockwise (**Fig 2E**), in 2 eyes as nasal anticlockwise (**Fig 2F**), and in 7 eyes as temporal distal shift (**Fig 2G**).

### Subanalysis in the RVS(+) group

**Table 3** shows the demographic data of the RVS(+) group. ΔPRAA was 0.06 ± 1.22 degrees, and the absolute value of ΔPRAA was 0.91 ± 0.80 degrees. Body weight, body height, and AL were significantly greater in the third grade than in the first grade (p < 0.0001, Wilcoxon signed rank test). PRAA was not significantly different between the first and third grades (p = 0.61, Wilcoxon signed rank test).

**Table 3:** PRAA of junior high school subjects of the RVS(+) group while they were in the first grade and the third grade.

	First Grade (12–13 years old)		Third Grade (14–15 years old)		*P* Value	
	Mean ± SD	Range	Mean ± SD	Range	
Subjects, n	42					
Sex (male/female)	19/23					
PRAA, degrees	133.8 ± 17.7	86.4–162.0	133.9 ± 17.8	86.4–162.1	0.61	

PRAA, Peripapillary retinal artery angle; RVS, retinal vessel shift; SD, standard deviation.

**Fig 4:**
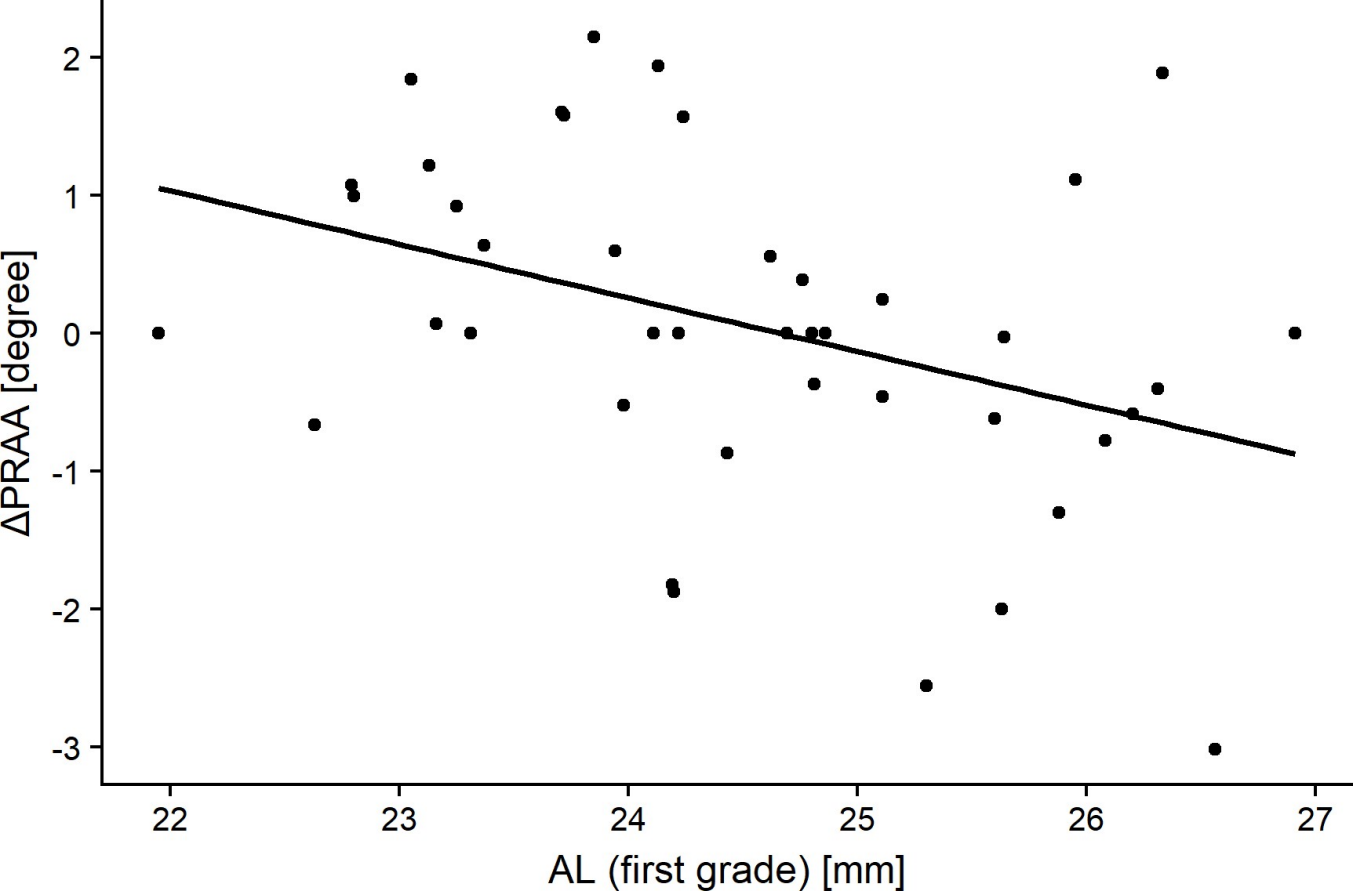
The relationship between the change in the peripapillary retinal arteries angle (ΔPRAA) and axial length (AL) in subjects when in the first grade. Significant correlations were obtained (R = -0.39, p = 0.012, Pearson’s product-moment correlation).

**Fig 5:**
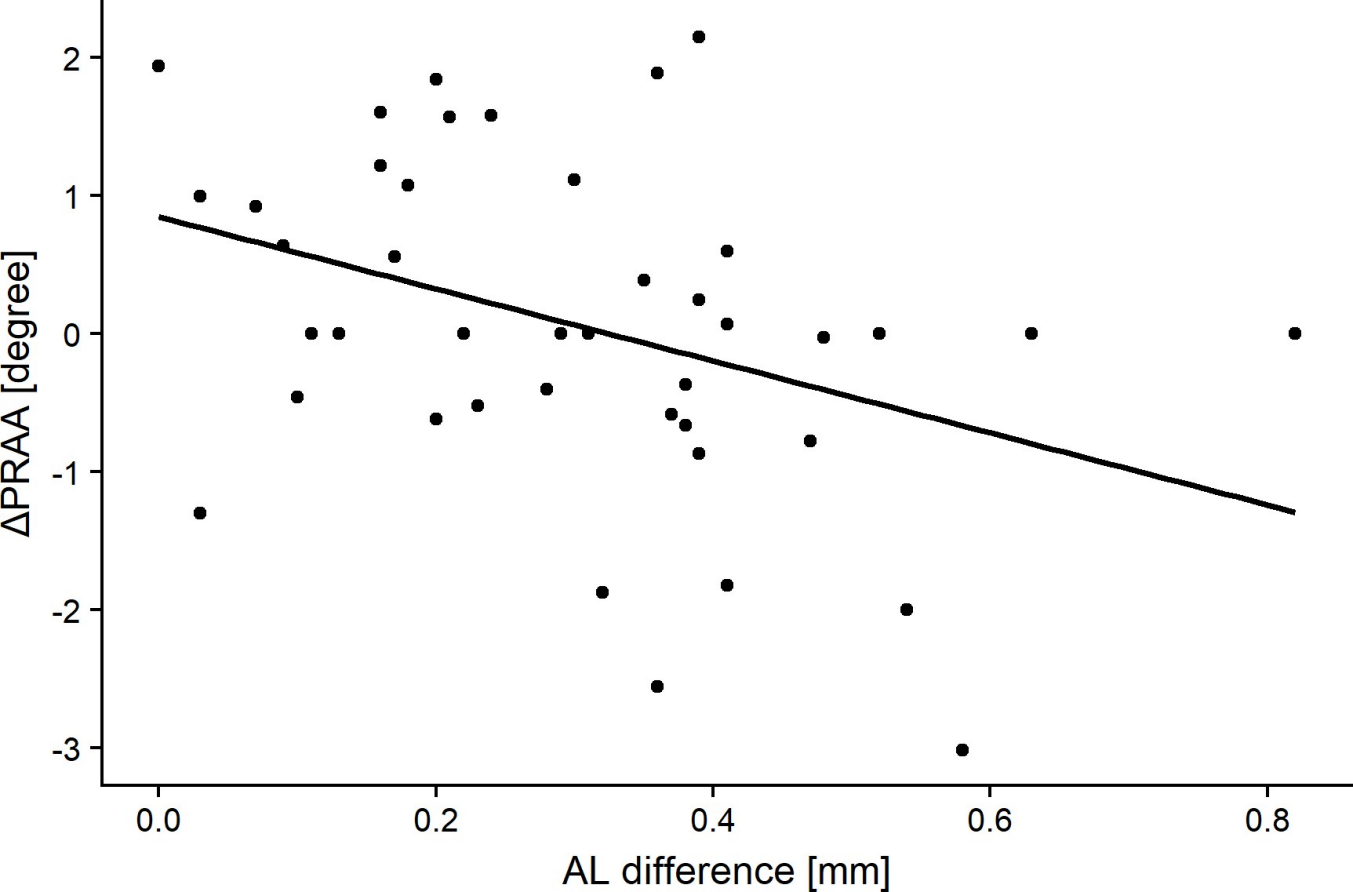
The relationship between change in the peripapillary retinal arteries angle (ΔPRAA) and the difference in the axial length of the subjects when in the third and first grades (ΔAL). Significant correlations were obtained (R = -0.37, p = 0.014, Pearson’s product-moment correlation).

ΔAL was significantly greater in eyes with negative ΔPRAA than in those with positive ΔPRAA (p = 0.03, Mann-Whitney U test). **Table 4** shows the relationships of ΔPRAA with sex, body height (first grade), body weight (first grade), ACD (first grade), AL (first grade), PRAA (first grade), Δbody height, Δbody weight, ΔACD, and ΔAL. ΔPRAA correlated negatively with AL (first grade) and ΔAL (R = -0.39 and p = 0.012, and R = -0.37 and p = 0.014, respectively, Pearson’s product-moment correlation, **Figs 4** and **5**). The optimal linear model for ΔPRAA was as follows: ΔPRAA = 9.48–0.2 (SE = 0.15, p = 0.12) × AL (first grade)– 0.26 (SE = 1.1, p = 0.02) × ΔAL– 0.06 (SE = 0.03, p = 0.07) × body weight (first grade) (AICc = 132.8).

**Table 4:** The relationships of the change in the PRAA with sex, body height (first grade), body weight (first grade), PRAA (first grade), AL (first grade), ACD (first grade), Δbody height, Δbody weight, ΔAL, and ΔACD in the RVS(+) group.

	Univariate analysis			Optimal model		
Variables	Coefficient	SE	*P* Value	Coefficient	SE	*P* Value
Gender	−0.060	0.38	0.88	N.S.		
Body height (first grade)	−0.048	0.030	0.12	N.S.		
Body weight (first grade)	−0.041	0.036	0.27	−0.063	0.034	0.072
PRAA (first grade)	0.0058	0.011	0.6	N.S.		
AL (first grade)	−0.39	0.15	0.012	−0.24	0.15	0.12
ACD (first grade)	−0.65	0.78	0.41	N.S.		
ΔBody height	−0.0018	0.036	0.96	N.S.		
ΔBody weight	−0.072	0.048	0.14	N.S.		
ΔAL	−2.61	1.02	0.014	−2.64	1.09	0.020
ΔACD	0.012	1.60	0.99	N.S.		

PRAA, Peripapillary retinal artery angle; AL, axial length; ACD, anterior chamber depth; Δbody height, body height change; Δbody weight, body weight change; ΔAL, AL change; ΔACD, ACD change; RVS, retinal vessel shift; SE, Standard Error; N.S., Not selected.

## Discussion

In the current study, fundus photography along with body height, body weight, and AL measurements were prospectively performed for two years in 161 eyes of 161 junior high school students. We observed RVS in 42 eyes (26.1%). Among these eyes, the patterns in 5 eyes were categorized as supra temporal clockwise, in 7 eyes as infra temporal clockwise, in 9 eyes as nasal clockwise, in 7 eyes as supra temporal anticlockwise, in 5 eyes as infra temporal anticlockwise, in 2 eyes as nasal anticlockwise, and in 7 eyes as temporal distal shift. AL was significantly longer in subjects from both groups while in the third grade than that during the first grade. The optimal model for the change in PRAA suggested that narrowing of PRAA was associated with larger AL in the first grade, greater elongation of AL, and greater body weight in the first grade.

In the current study, body height and weight were significantly greater in the third grade as compared to that in the first grade, as expected. In addition, AL was significantly longer in the third grade as compared to that in the first grade with average differences of 0.30 mm and 0.29 mm in the for RVS(+) and RVS(−) groups, respectively. We have recently reported that AL increases even in adults (mean age: 61 years old, range 32–88 years) by 0.031 mm in 5 years on an average [27]. The observation period in the current study was 2 years, suggesting that AL elongation between the ages of 12 and 14 was much greater than that during adulthood [27]. Greater AL elongation was associated with longer AL (**Table 2**). This relationship between AL elongation and longer baseline AL implies that elongated eyes may have a weaker collagen structure and tend to elongate easily. This current result coincides with previous reports of high myopia at baseline being a risk factor for myopic progression [28,29]. Our previous study showed an opposite trend in a glaucoma cohort [27]. This may be because the previous study consisted of adults with complete growth of eyeballs; thus, the elongation of an eyeball in the studied cohort was not identical to the growth of an eyeball in children.

In the current study, no significant differences in AL and ΔAL were observed between the RVS(+) and RVS(−) group. This may be because of the manifold mechanisms involved in eye growth. In general, eye growth occurs in the following three patterns: 1) proportional growth of all the dimensions leading to a spheroidal shape; 2) predominant elongation of the anterior-posterior axis; or 3) a local elongation of the posterior pole by a posterior staphyloma [30,31]. In patterns 2 and 3, the changes can make the curve of the arcade vessel steeper and narrower [7,30,31], while this shift may not occur in pattern 1) because of the proportional and global growth of an eye.

The current study suggested that there were various subtypes in RVS; 5 eyes were categorized with supra temporal clockwise pattern, 7 eyes with infra temporal clockwise, 9 eyes with nasal clockwise, 7 eyes with supra temporal anticlockwise, 5 eyes with infra temporal anticlockwise, 2 eyes with nasal anticlockwise, and 7 eyes with temporal distal shift. The entire mechanism underlying each subtype is unclear; however, they may be related to the variation in the ocular shape changes. The supra temporal anticlockwise (**Fig 2D**) and infra temporal clockwise RVS (**Fig 2E**) could be explained by the retinal artery shift in relation to the predominant elongation along the anterior-posterior axis. This is because the retina in the equator zone is predominantly stretched in an oval shaped eyeball, and RVS is moved toward the papillo-macular bundle. In contrast, local/unproportional eye elongations occur not only in eyes with high myopia or pathological myopia. For instance, myopic maculopathy such as tessellated fundus and myopic chorioretinal atrophy can develop locally around posterior pole with moderate myopia [32]. The temporal distal and infra temporal anticlockwise RVS might be reflecting such focal elongation of the eye toward the inferior direction. The supra temporal clockwise (**Fig 2A**), infra temporal anticlockwise (**Fig 2B**), and nasal clockwise (**Fig 2C**) RVS coincide with the movement of the optic fissure closure [33], implying that the structural changes correspond to the optic fissure closure may still be present even at the growth period. The understanding of RVS may help us to elucidate the underlying mechanism of myopic retinal stretch.

In the RVS(+) group, the average absolute value of ΔPRAA was 0.91°. The PRAA value was not significantly different from that in the first grade, because of the mixture of minus (narrowing; 16 eyes with -1.11 degrees in average), plus (widening; 18 eyes with 1.13 degrees in average), and zero (8 eyes) in values. It is noteworthy that ΔAL were greater in eyes with negative ΔPRAA and ΔPRAA was significantly associated with ΔAL (see **Table 4** and **Fig 5**), suggesting that PRAA becomes narrow with the elongation of an eye. We have recently proposed PRAA to evaluate the retinal stretch associated with the elongation of an eye based on the results of a cross-sectional investigation [7,10,11]. This is supported by the current results obtained from a prospective and longitudinal cohort on children of growing age (childhood). The optimal model for ΔPRAA also included AL (first grade), suggesting that longer AL in the first grade was associated with the narrowing of PRAA (see **Table 4** and **Fig 4**). This may be because elongated eyes in the first grade are likely to exhibit further elongation of an eye, and the retinal structural change, as represented by PRAA narrowing, is predominantly observed in such eyes. The current result also indicated that increase in body weight was also related to further PRAA narrowing. In contrast, body weight at the first grade was negatively correlated to ΔAL. A previous cross-sectional study has reported that body weight was positively correlated to AL in Japanese elementary school children [34]. Other previous studies have suggested a positive and significant correlation between AL and high cholesterol intake [35,36]. However, the underlying mechanism between body weight and eye elongation remains unclear, suggesting the need for the further investigation elucidating the relationship between body weight (and its gain) and eye elongation. It is important to understand not only the mechanism of the development of myopia, but also ocular pathologies. Myopia is an established risk factor for the development of glaucoma [12–15], and we have reported that AL elongation is associated with the progression of glaucomatous visual field damage in adults [27]. On the other hand, interestingly, the previous study reported that the increase in the AL was associated with slower progression of glaucomatous visual field damage in inferior hemifield, probably because AL elongation reduces the stress to lamina cribrosa [27]. Hence, it would be of interest to further investigate the relationship between the elongation of AL in childhood and the future development of glaucoma in a future study.

There are certain limitations of the current study. We superimposed the photos manually using the optic disc and supra temporal fine retinal vessels as reference points in the current study. We also tried different methods to superimpose the images, such as using fovea-centered images and choosing other vessels as landmarks. It turned out that the method adopted in the study (superimposing the images by adjusting the fine vessels on the optic disc and vessels in the temporal side of the optic disc) resulted in the best quality in evaluating RVS. Moreover, we tried built-in program of ImageJ software called Extract SIFT Correspondences technique, which did not work. We need to develop an automatic superimposing program to standardize the analysis of RVS and PRAA in the future. Second, the number of participants enrolled in the current study was relatively small. Although we found 7 subtypes in the current study, there may be other types of RVS. Other variations may be found using MRI with longer follow-up period. In addition, the study population was made up of Japanese volunteers who are known to belong to one of the most myopic group in the world [37]. Thus, our results might not necessarily hold for non-myopic individuals of other ethnicities. Moreover, although myopic change may cause ocular structural changes in a longer duration, the follow-up period was limited to two years. A study with a larger sample size from different ethnic groups with a longer observation period will enable us to compare the relationship between PRAA and AL in each subtype in detail.

In sum, about 26.1% of the eyes of junior high school subjects who were in the first grade (12- or 13-years old) exhibited RVS in two years. There were several different changing patterns of RVS in this period, with most RVS patterns being unbalanced or localized. RVS was associated with AL elongation, suggesting that eye elongation may be focal and/or irregular even in normal eyes.

## Supporting information

S1 File The data analysed. (CSV)
